# Before the Lords' Committee

**Published:** 1890-05-24

**Authors:** 


### Before the Lords' Committee.

(By Our Special Commissioner.)

It has been a matter of some difficulty to determine the best way of treating the proceedings before the Lords' Committee so as to make them easily understood by those they most concern. It is manifestly impossible to report verbatim voluminous evidence occupying from three to four columns of the *Times* newspaper. Besides, the quality of the evidence depends not only upon the witness but also to an extent upon the period at which he is examined. Much evidence must necessarily be tendered and many details given which would be altogether out of place in our columns. On the other hand much has already been given which deeply concerns not only the welfare but the very existence of the institutions mentioned, which has so far found no place in the columns of the press. In these circumstances we have determined to undertake the labour of condensing each witness's evidence by eliminating everything which is not material, and to assist the reader by classifying every important point. We would especially direct the attention of the managers of the hospitals to the importance of their sending some accredited representative to the meetings of the Lords' Committee so that they may at once become aware of any charges and statements which affect them, and which ought to be dealt with officially by a properly-accredited representative before the termination of the Committee's labours. Unless this is done, many excellent institutions may find themselves in the unfortunate position of being accused of somenegligence or enormity, of the existence of which they had no idea, and a knowledge of which may only reach them after the proceedings are published.

### A Caution to the Press and Public.

It must not be forgotten that the evidence is very often highly technical, and consequently difficult to follow by a person not acquainted with hospital administration. The newspaper reporters have therefore a most difficult task to perform, especially as they have to make a selection from a long statement by a witness, and to condense it into a column, or half a column, as their respective editors may direct. It follows that errors and misunderstandings must appear in these reports. A notable instance of this has already occurred. Dr. Steele was reported to have stated that he strongly objected to special hospitals, and especially to special hospitals for children. This remark has caused a good deal of surprise and comment, as well it might do, because it is well known that no special hospital does better work, nor is its existence as a separate institution more generally admitted to be necessary than a hospital for sick children. What Dr. Steele really said in reply to the question, "Are you in favour of special hospitals for children?" was, "With Great Ormond Street before me, I should not like to say that I was not favourable to them. I think hospitals for children very good institutions." This is no doubt a typical example of what we may expect in the course of the inquiry, and we reproduce it here to emphasize and enforce the caution we have thought it necessary to give.

### Colonel Montefiore.

Colonel Montefiore's evidence was remarkably able, and on the whole he stated the case of the C.O.S. against the present system of hospital administration with moderation and force. There are, however, some remarkable errors which he failed to correct in his re-examination on the 15th inst. Thus he stated that the endowed hospitals, St. Bartholomew's, Guy's, and St. Thomas's, are entirely for general diseases, from which it may be inferred that he meant that they did not admit special diseases. As a matter of fact, each of these hospitals has numerous special departments in charge of specialists, who have a certain number of beds allotted to them for the purpose of treating cases belonging to the various specialities they individually represent. He further stated that the Provident Dispensary was exactly what is known in the country as a sick club. A truly remarkable statement, which is, of course, inaccurate and misleading. Again, he stated, that well-conducted Provident Dispensaries would distinctly have a wage limit, as without it, they would create as much abuse as exists at present at the Hospitals. Now it is the opinion and experience of the best managed Provident Dispensaries that the wage limit is often unfair in its incidence, and that for practical purposes it has little effect in limiting the members to really deserving cases. Again, he held that the introduction of the threepenny fee at Guy's Hospital had tended to diminish the number of applicants for out-patient relief. This we believe to be quite erroneous. He further expressed the opinion that the competition amongst Hospitals was so keen that they spent year by year sums considerably larger than their average income would justify, and were thus driven to resort to all manner of contrivances to meet their liabilities. This is an unintentional but undoubted and complete misrepresentation of the facts. No doubt unintentionally, he did a great deal of injustice to the Hospital for Epilepsy and Paralysis, Portland Terrace, Regent's Park, when he classed it with the West-End Hospital for Epilepsy, in Welbeck Street. He was further inaccurate in stating that University College Hospital was fairly close to Welbeck Street, unless a mile of distance can be held to be correctly described as "fairly close." He stated, too, that the clinical clerk is supposed to be just qualified, whereas, of course, most clinical clerks are fourth year's men, though it sometimes happens that the post is given to qualified men also. He was wrong again in stating that the Metropolitan Poor Law Infirmaries, when first established, were used as medical schools, the fact being that a clause permitting clinical instruction was inserted in the original Bill, but eliminated before the Act was passed. We further cannot agree with him that the wave of prejudice is being revived in the minds of the poor against the hospitals and poor law infirmaries because unclaimed and paupers' bodies are used for dissection. It was inaccurate to define a voluntary hospital as one entirely supported by voluntary subscriptions, seeing that the voluntary hospitals of this country have at present invested and other properties to the value of from seven to ten millions sterling. We propose to deal with the suggestions made by this witness on behalf of the C.O.S. in a subsequent article, but we cannot refrain from expressing much surprise at the twelfth of these proposals. It is that the Hospital Saturday and Sunday Funds shall in future make their grants on the award of or in consultation with the General Council which it is proposed to establish to control the whole of the metropolitan medical charities. Anything better calculated to destroy these funds it would be difficult to imagine.

### Accusations against Particular Institutions.

It may be useful to give the names of those of the better known hospitals against the administration of which accusations have been made. They are the Hospital for Children, Great Ormond Street, where it is stated that patients are kept waiting from 8.30 a.m. till 3 p.m.; the Great Northern Central Hospital, where a patient waited all the afternoon for a certificate to be signed, and was then told it was too late. At the Evelina Hospital for Children one patient is said to have waited from 9 a.m. to 8 p.m. before being attended to; another one from 2 p.m. to 8 p.m.; another from 11 to 3—the surgeon then refusing to examine the child. It was further stated to be a common practice at this hospital to keep patients waiting from 9 a.m. till 5 p.m. Similar complaints are made against St. Thomas's Hospital and the Brompton Hospital for Consumption. At the London Hospital a man is stated to have been kept sitting in the crowded waiting-room of the out-patient department with a suspicious-looking skin disease. When the porter called the surgeon's attention to it, he summoned the students with the words, "Come along, boys; here's a chance. We don't get a case of small-pox every day." This apparently happened a considerable time ago. At Guy's Hospital, a woman and her two children, who were suffering from whooping cough, were seated amongst a lot of patients, several of whom were children, from eleven a.m. to three p.m., or later. At St. Bartholomew's Hospital, a boy in whom Colonel Montefiore was interested was found to have the measles, which disease the witness contracted from the lad in question. We hope the hospital authorities in each of these cases will communicate with the C.O.S., and take care to give evidence as to the special cases referred to, and the general practices which they are stated to illustrate.

### Overlapping.

The following remarkable cases of overlapping, showing the necessity for inter-communication and co-operation amongst hospital authorities, were given: A woman was treated for six weeks at the City Road Chest Hospital in June, 1889. In November, 1889, she went to the Brompton Hospital for Consumption for three months. In March, 1890, she was admitted as a patient to St. John's and St. Elizabeth's Hospital. In another remarkable case a patient is stated to have been four months in an invalid asylum during 1888-89. In the latter year she moved to St. Andrew's Convalescent Home at Folkestone, and subsequently to the Convalescent Home at Southend. In February, 1890, she was admitted to the Hospital for Consumption in the City Road, where she remained a month, subsequently finding her way to the Brompton Hospital for Consumption, where she stayed for six weeks, when the doctor stated that she had no organic mischief, but was merely suffering from weakness. Another person is stated to have attended as a patient at the out-patient department of St. Thomas's Hospital for a swelled knee, and at the same time to have received treatment at the out-patient department of St. Bartholomew's Hospital for a tumour in his side. One day when he found the out-patient department of St. Bartholomew's closed when he arrived, he transferred his affections to Guy's, and there received treatment. Instances were given of the way in which patients abuse the Samaritan Funds of the hospitals. Thus, the parents of one patient, a child, succeeded in procuring an instrument valued at upwards of three guineas from the National Hospital for Epilepsy and Paralysis. When this apparatus was broken, instead of having it repaired the parents took the child to the London Hospital, where they obtained a certificate for a new instrument, but on this coming to the knowledge of the C.O.S., they found the old one, had it repaired, and it served its purpose admirably. Another case, that of a man, is quoted, who obtained an artificial leg from one of the societies, but not liking its shape, he calmly put it by and started off to beg letters to enable him to procure one of a pattern which suited his fancy better. His attempt was, however, discovered, and he was then made to use the limb he already possessed. We have selected these instances by way of illustration and in proof of the undoubted fact that some instruction as well as amusement may be derived from a visit to the committee room of the House of Lords.

### A Hint to Hospital Authorities.

It is a great pity that the full evidence given from day to day before the committee is not published. We believe the cause to be the delay which the necessary corrections entail. We are of opinion, however, that having regard to the importance of the inquiry to the hospitals generally it would be distinctly beneficial if Lord Sandhurst could make arrangements for the evidence to be on sale at a small cost. Failing this, and, indeed, in any case, we would strongly recommend hospital managers to arrange that some member of their committee or their secretary should attend each meeting of the Lords' Committee. The Committee meets twice a week, on Mondays and Thursdays, and it should not be difficult for the hospital committees to arrange a *rota* in such a way as to ensure that at least one representative will always be present whenever the Committee sits. By this means they would be able to obtain the earliest information of any charges, complaints, or other points which may arise from time to time affecting their interests and credit, and so secure that every statement of the kind is thoroughly investigated, and that the Committee shall never be misled as to the actual facts and circumstances of every case submitted for their consideration. We shall do our best to reproduce every material point of this kind which may arise for the information and guidance of the hospital committees. We have no doubt that they will welcome our co-operation in this respect, and that many of them will adopt some such proposal as we have thought it our duty to make in their own interests.

*(To be continued.)*

